# Primary care management of diabetes in a low/middle income country: A multi-method, qualitative study of barriers and facilitators to care

**DOI:** 10.1186/1471-2296-8-63

**Published:** 2007-11-09

**Authors:** Hugh Alberti, Nessiba Boudriga, Mounira Nabli

**Affiliations:** 1 Institute of Health and Society, Newcastle University, 21 Claremont Place, Newcastle upon Tyne, UK, NE2 4AA; 2 DSSB (Direction du Soins de Santé de Base), 31 Rue Khartoum, Tunis, Tunisia

## Abstract

**Background:**

The management of patients with diabetes mellitus is complex. Some research has been done in developed countries to attempt to determine the factors that influence quality of care of patients with diabetes: Factors thus far postulated are usually categorised into patient, clinician and organisational factors. Our study sought to discover the main barriers and facilitators to care in the management of diabetes in primary care in a low/middle income country.

**Methods:**

A qualitative study, based on reflexive ethnography using participant observation, semi-structured interviews of clinicians (10) and group interviews with paramedical staff (4) and patients (12) in three purposively sampled health centres, along with informal observation and discussions at over 50 other health centres throughout Tunisia. A content analysis of the data was performed.

**Results:**

Over 400 potential barriers or facilitators to care of patients with diabetes in primary care in Tunisia emerged. Overall, the most common cited factor was the availability of medication at the health centre. Other frequently observed organisational factors were the existence of chronic disease clinics and clinicians workload. The most commonly mentioned health professional factor was doctor motivation. Frequently cited patient factors were financial issues, patient education and compliance and attendance issues. There were notable differences in the priority given to the various factors by the researcher, physicians, paramedical staff and the patients.

**Conclusion:**

We have discovered a large number of potential barriers and facilitators to care that may potentially be influencing the care of patients with diabetes within primary care in Tunisia, a low/middle income country. An appreciation and understanding of these factors is essential in order to develop culturally appropriate interventions to improve the care of people with diabetes.

## Background

The management of people with diabetes mellitus is complex. Good control significantly reduces the risk of complications [1,2], yet studies from around the world consistently demonstrate inappropriate variations in care [3-5]. Efforts to improve the quality of care should be informed by knowledge of which factors influence care and how they act as barriers or facilitators. Previous research around diabetes care has grouped such factors under the general headings of patient, health professional and organisational factors [6,7]. Ideally, quality improvement efforts should be underpinned by more specific knowledge of modifiable factors amenable to change. Furthermore, factors identified in previous work from Europe and North America may not be transferable to other cultures. Few studies have been reported from the developing world, despite the knowledge that 80% of all chronic disease deaths worldwide now occur in low/middle income countries [8].

Tunisia is a country of 10 million inhabitants situated on the North African coast. The prevalence of diabetes mellitus has significantly increased over recent decades to around 10% [9]. In response, the Tunisian Ministry of Health initiated a national program to improve the management of hypertension and diabetes in primary care with the overall objective of ensuring quality, standardised, regular care in order to reduce complications [10]. The program comprises eight components: Training of primary health care doctors; development of standardised, national guidelines; initiation of chronic disease clinics; screening; provision of essential medicines and screening materials; local supervision of the program; public health education; and development of data collection systems.

Tunisia's gross national income is $2.650 per capita placing it 102nd in a list of 208 countries in the world according to the World Bank (2004 data) [11]. Adult literacy rate is high relative to nearby countries at 74% of adults and total life expectancy is 73.4 years (2004 data) [12]. Tunisia has two major health sectors, public and private, said to be complementary rather than competitive. Patients may choose whether to attend primary or secondary, public or private care. The majority of Tunisians in employment pay for health insurance, which covers most, but not all, of health expenses within the public sector and some aspects of the private sector.

Our study aimed to identify specific barriers and facilitators to improving the primary care management of diabetes in primary care in Tunisia.

## Methods

We used a multi-method qualitative approach given its recognised value in investigating diabetes care elsewhere and the known complexity in determining barriers and facilitators to care [6,7]. The underlying conceptual framework was based on reflexive ethnography [13]. Throughout the study the terms 'diabetes care' and 'management of diabetes' are used interchangeably and refer to all aspects of patient care including metabolic and blood pressure control as well as access and monitoring issues.

The Tunisian Ministry of Public Health granted permission for the study.

### Setting and Participants

The study population included people with diabetes and health professionals providing diabetes care in public sector, primary care health centres in Tunisia. Participant observation, semi-structured interviews and group interviews were undertaken at three purposively sampled health centres, supplemented by informal observation and discussions at 48 randomly selected health centres.

### Participant Observation

HA (the 'researcher') visited the three health centres weekly for six-months each. He observed all events at the health centre; consultations with the doctor and nutritionist, the various roles of the nurses, the pharmacy, the waiting room and the reception areas. Observations and discussions witnessed relevant to the research question were recorded in condensed or expanded form in the researcher's fieldnotes [13]. The fieldnotes also detailed the researcher's personal reactions to events and changes in views over time.

### Interviews

Semi-structured interviews were conducted with the lead physician of each of the three health centres and seven other key informants. The interview schedule was designed around the central question: "What are the patient, clinician and organisational factors that influence the care of patients with diabetes in Tunisia?" One paramedical staff group interview and four patient group interviews were conducted at each of the three health centres based on an open interview schedule developed around the central question: "What do you think about the management of patients with diabetes here?" A total of 15 paramedical staff, 5 men and 10 women, participated in the staff group interviews and 40 patients, 15 men and 25 women, participated in the patient group interviews. HA conducted all the interviews in Tunisian Arabic, audiotaped them and later simultaneously transcribed and translated them into English. A Tunisian English teacher reviewed and corrected all transcripts by listening to the original recording whilst reading the draft transcription. Written, informed consent was obtained for the individual interviews and oral, informed consent for the group interviews.

### Other data sources

Other data sources included additional participant observation by HA at various other locations during the four-year project: Visits to 48 randomly selected health centres throughout Tunisia, attendance at 19 relevant meetings related to diabetes care and prolonged exposure in the department that coordinates the national program of diabetes care in Tunisia. All relevant observations and discussions were recorded in the fieldnotes and incorporated into the data analysis.

### Data Analysis

Transcripts of all interviews and fieldnotes from participant observation formed the raw data for analysis. The processes of sampling, data collection and data analysis were continuous and iterative. A content analysis [14] was conducted using the software computer program NVivo to systematically code and classify data into barriers and facilitators to care according to the predetermined categories of patient, health professional and organisational factors. During analysis the codes were regularly reviewed and re-grouped or re-classified according to new data. The frequency that each factor was coded was noted in addition to the source (i.e. doctor, paramedical staff, patient, researcher or other).

### Quality assurance of data analysis and interpretation

The consistency (reliability) and confirmability (validity) of data analysis and interpretation were assessed using five widely used techniques [13,15]. Firstly, a summary of the interviews of health professionals was discussed with the interviewee at a later date to validate the findings. Additional comments were included in the transcripts (respondent validation). Secondly, the results were triangulated with other data sources within the study (triangulation). Thirdly, throughout the data collection and analysis we remained sensitive to the ways in which the researcher and the research process shaped the findings, including the role of prior assumptions and experience (reflexivity). Fourthly, in addition to time spent in each health centre undertaking formal participant observation, HA spent four years in total undertaking the project (prolonged engagement in the field). Fifthly, all decisions made regarding the methods of data collection and analysis were clearly explained in the fieldnotes and are available for further reflection or inspection (clear audit trail).

## Results

412 different, specific, barriers or facilitators to care of people with diabetes in primary care in Tunisia emerged within the categories of patient, health professional and organisation.

### Patient factors

The ten most frequently mentioned patient factors are listed in Table 1. Financial constraints were often mentioned by doctors and patients and were always described as barriers to care: "If a person doesn't have the money to register or to go some other place, it's a problem. He has to wait on God. He has nothing to do. It's a question of the financial situation. One day a person might have 5 dinars (≈ $4), the next day they have nothing" (patient, group interview 6). Patients and health professionals described financial barriers influencing various aspects of the patients' care, such as attending the health centre, travelling to the local hospital for blood tests and appointments and purchasing medications if unavailable at the health centre.

**Table 1:** The number of passages coded for the ten most frequently mentioned patient factors and their sources

***Patient factors***	**Total**	**Researcher**	**Doctors**	**Paramedical staff**	**Patients**	**Others**
Financial constraints	**69**	7	26	5	24	7
Compliance with medication	**68**	20	23	10	12	3
Compliance with diet	**63**	9	19	13	17	5
Patient education	**57**	8	24	4	7	14
Gender issues	**52**	10	13	9	14	6
Use of alternative medicine	**43**	3	17	5	15	3
Attendance at clinics	**35**	13	11	8	0	3
Compliance with referrals	**27**	9	14	3	0	1
Knowledge of diabetes	**27**	13	2	1	9	2
Also attend other places	**23**	13	4	4	0	2

Others: Directors, doctors at the national program centre and other key informants

Poor patient compliance was a frequently mentioned barrier to care, encompassing adherence to diet, medications, blood tests and referrals. Patients and paramedical staff considered dietary compliance to be the most relevant problem whereas doctors were more likely to mention medication compliance: "I don't follow it (a diet) at all...I just eat normally" (patient, focus group 11). "There are patients who stop their medicines when they feel like it" (doctor, semi-structured interview 2). Patients and health professionals often quoted financial reasons as the cause of poor patient compliance.

Clinicians often cited patient education as a problem in the management of patients and seemed to blame their patients for being poorly educated. However, staff and patients acknowledged the steps being made to address this issue and many were positive about the improvements achieved: "They (the doctors) are really good with us and they talk to us and they guide us... and they tell us we must continue our medications and never let them run out" (patient, group interview 5).

The influence of gender on attendance at health care facilities emerged as a major theme: 62% of patients consulting with diabetes are women despite the similar prevalence rates^9^. The reasons given were that men tended to under-attend due to work commitments, attending other health care facilities and a perception that their health was not important. Women were considered to be "iller" and therefore required more care, but were also thought to over-attend: "For women her time is always staying at home. She always wants to come, especially those that don't have the means for leisure activities. She attends the centre and gathers and chats with her friends" (female nurse, staff group interview 1).

In Tunisian Arabic, herbal medication is literally translated as "Arabic medicine" in contrast to pharmaceutical medications, "French medicines". Clinicians most often mentioned their use. It was known that many older patients took them, but their use was not perceived to be a barrier to care: "She says that as long as its not harmful she lets the patients continue taking it" (fieldnotes, discussion with a doctor, centre 27).

### Health Professional factors

The ten most frequently mentioned health professional factors are listed in Table 2. Doctor motivation was the most important health professional factor to emerge, most frequently alluded to by the doctors themselves. Motivation was seen as a collective term covering multiple issues such as the doctors' interest, intentions and professional conscience. Many doctors and managers saw doctor motivation as the most important influence on the quality of care given: "For the management to be good regarding the doctors its necessary that they are motivated. They must know the things regarding diabetes and want to know more about it... he must put himself in the place of the patient, which is a little difficult, but its necessary to be a good doctor" (doctor, semi-structured interview 1).

**Table 2:** The number of passages coded for the ten most frequently mentioned health professional factors and their sources

**Health Professional Factors**	**Total**	**Researcher**	**Doctors**	**Paramedical staff**	**Patients**	**Others**
Motivation of doctors	**102**	32	41	1	0	28
Doctors time with patients	**43**	13	8	2	18	2
Role of nurses	**42**	31	3	2	1	5
Shortage of paramedical staff	**36**	4	19	9	0	4
Doctors work time	**36**	18	7	1	1	9
Teamwork	**29**	16	4	8	0	1
Shortage of specialists	**29**	2	21	1	0	5
Lack of feedback from specialists	**28**	14	12	1	0	1
Placement of doctors	**28**	8	15	0	0	5
Lack of doctors training	**27**	5	14	0	0	8

Others: Directors, doctors at the national program centre and other key informants

Themes around the topic of the doctor/patient relationship were often observed, and notably, the time spent by the doctor with the patients. Very different perspectives emerged from the various sources. The researcher noted the short time spent with each patient by the clinicians. The clinicians acknowledged that the large number of patients consulting prevented them from giving sufficient time to the patients: "He said that...patient's don't even know what the complications of diabetes are, and doctors don't have time to explain everything to them" (fieldnotes, discussion with a doctor, centre 17). In contrast, the patients never spontaneously mentioned lack of time as a problem, and when asked directly they usually stated that they had sufficient time with their doctor.

The role of health professionals, particularly the nurses, was a factor often noted by the researcher, probably due to the perspective of having worked in a different context in the United Kingdom. One area often alluded to was the measurement of blood pressure: Whose responsibility was it? "He said there are not enough doctors for the number of patients; for example, nurses have to do medical acts, doctors jobs, such as taking blood pressures, since there are so many patients" (fieldnotes, discussion with a doctor, centre 20).

Lack of nurses, dieticians and primary and secondary health care doctors were all mentioned as possible barriers to care, particularly by the professionals themselves: "One of the big problems is the problem of specialists, there are not enough ophthalmologists, there are not enough nephrologists, there are not enough cardiologists, to look after the complications of patients with chronic diseases" (doctor, semi-structured interview 3). In contrast, patients did not refer to shortage of staff as a significant problem.

### Organisational factors

The ten most frequently mentioned organisational factors are listed in Table 3. Lack of availability of medication at the health centres was the most frequent factor cited in all categories. The problem varied between regions and between centres but where it was a problem, it was seen as the most significant issue: "Shortage of medicines is the only problem...when they (the medicines) are available all the patients are happy and there are no problems" (patient, focus group 11).

**Table 3:** The number of passages coded for the ten most frequently mentioned organisational factors and their sources

**Organisational Factor**	**Total**	**Researcher**	**Doctors**	**Paramedical staff**	**Patients**	**Others**
Availability of medication	**157**	44	47	15	35	16
Use of chronic disease clinics	**96**	27	49	7	2	11
Clinician workload	**77**	22	37	6	3	9
Availability of HbA1c testing	**42**	11	23	0	0	8
Distance to specialists	**40**	6	17	5	7	5
Waiting time at health centre	**39**	21	5	0	10	3
Waiting time to see specialist	**37**	5	16	1	10	5
Organisation of centre	**36**	20	2	4	8	2
Problems with the managers	**35**	7	14	7	2	5
Lack of resources	**32**	3	14	5	0	10

Others: Directors, doctors at the national program centre and other key informants

The national program of diabetes care has encouraged the introduction of weekly chronic disease clinics. Many doctors attribute improvements in quality of care to these clinics: "She says the care has improved compared to the past, partly due to the chronic disease clinics which allow the doctors to focus on the chronic patients" (fieldnotes, discussion with a doctor, centre 37).

A heavy workload due to the large number of patients consulting at the health centres was a barrier to care cited particularly by the doctors: "He said its busy: 60–100 patients a day and sometimes the doctor has to see them all by himself" (fieldnotes, discussion with a nurse, centre 27).

Widespread lack of availability of HbA1c testing was perceived as an important barrier to care by the researcher and by doctors. In most regions HbA1c testing is not available in primary care, although this potentially important barrier tended to only be mentioned by younger doctors: "He realises that lack of HbA1c is a big problem... he says the public laboratories don't do them so people have to go to private laboratories, so only professionals get it done" (fieldnotes, discussion with a doctor, centre 8)

A number of issues relating to secondary care were seen to impinge on the care of patients with diabetes at the primary care level, linked to the fact that some of the requirements of routine diabetes care, such as eye examinations, are reliant on secondary care. The most common barriers to care raised, particularly by clinicians, were the distance patients had to travel to reach secondary care and the waiting times at the local hospitals: "The doctor asked the patient, "if I send you to (Town X) for an eye check will you go? Patient said no, it was too far" (fieldnotes of observing consultations, centre 26).

## Discussion

Our study has identified numerous, specific barriers and facilitators to care that may potentially be influencing the management of diabetes within primary care in Tunisia, a low/middle income country.

### Factors influencing diabetes care

The large number of specific barriers and facilitators to care to emerge is striking, but perhaps not unexpected, given the complex nature of diabetes care. The most important factors to emerge were; availability of medication, doctor motivation, chronic disease clinics, financial constraints, patient compliance and attendance and clinician workload. Many of the key factors identified have been reported from studies in developed countries with some notable exceptions.

Firstly, the primary importance placed on the availability of medication at the health centres. This finding in our context may not be surprising given that medication is provided free for patients on payment of a minimal consultation fee. This correlates with financial constraints being the most important patient-related factor. In low/middle countries, financial aspects continue to strongly influence the care of patients, especially those with chronic diseases [16].

Secondly, and arguably the most surprising finding of our study, was the significance placed on doctor motivation, and particularly how much more important it was considered to be relative to clinician training. Although the attitudes and beliefs of clinicians have been highlighted in some previous work in developed countries [17,18], it appears to be a neglected factor relative to other issues. Theories of human behaviour may offer useful means of understanding factors such as motivation and designing strategies to change practice.

Thirdly, the influence of gender on attendance at health care facilities and the use of herbal medicine have seldom been reported as factors influencing diabetes care.

In contrast, our other major findings correlate with work from developed countries: Organisation-related factors such as the use of chronic disease clinics [19] and clinician workload [6]; patient-related factors concerning compliance, education and attendance [7,18] and health professional factors such as the role of doctors and nurses [20] and the length of consultations [21] in primary care.

In addition, the notable differences in significance attached to the various factors by the participants involved correlates with findings from developed nations that patients and healthcare professionals have diverse perspectives towards diabetes care [17,18].

Smaller, quantitative studies in Tunisia by ourselves and others have previously suggested a small number of factors that may influence the care of people with diabetes in Tunisia: Namely, the use of disease-specific medical records, the distance patients need to travel to receive care, milieu of the health centre and physicians interest in diabetes [22-24]. Although all of these potential factors emerged in this larger, more in-depth, national study, other factors appear to be more important. We have tested many of the hypotheses generated in this study in a parallel, quantitative project [25]. The factors found to be significantly and independently associated with quality of care indicators were very similar to the most commonly cited factors from the content analysis (regional affluence, doctor motivation, use of chronic disease clinics, younger patient age and availability of medication).

It is encouraging to note that many of the key factors to emerge are being addressed in the Tunisian national program of diabetes and hypertension management, such as the use of chronic disease clinics, public health education and the provision of essential medicines [10].

### Strengths and limitations of the study

Our qualitative approach has enabled the identified barriers and facilitators to care to be described and explored in a way that would not have been possible with quantitative methods. The rigorous application of multiple qualitative methods ensured a broad range of opinions, but the utility of the study depends not on the generalisability of their results but their ability to generate hypotheses and explore them in some depth. Our study is one of the first to be reported from a low/middle income country and thus our findings are more likely to be transferable than previous work in developed countries. An acknowledged potential bias of our study is the central role played by the researcher in the participant observation, interviews and data analysis. In addition, the lead researcher originates from outside of Tunisia (England); although being an outsider has potential advantages such as increased objectivity [13], this may have prevented him from being fully 'sensitive' to the way in which he influenced the research findings (reflexivity).

## Conclusion

We re-iterate that this is an exploratory, qualitative study with the aim of generating hypotheses and exploring the barriers and facilitators to care of people with diabetes in Tunisia. The use of multiple methods has identified numerous factors that warrant further investigation in Tunisia and other similar low/middle income countries. The ultimate goal is to determine the most influential barriers factors that are potentially amenable to change in order to improve the care of patients: Thus far, doctor motivation appears to be the most important factor amenable to intervention. Further studies are underway in Tunisia to identify the exact role of doctor motivation in diabetes care in order to design and develop culturally appropriate interventions. It is unlikely that such extensive and in-depth studies as ours can be undertaken in every context, particularly less affluent countries. We would therefore recommend that clinicians, managers and health policy makers take our results into consideration in order to develop and implement culturally appropriate, quality improvement interventions in other low/middle income countries.

## Competing interests

NB and MN are co-ordinators of the national program of diabetes and hypertension management in Tunisia.

## Authors' contributions

HA had the initial idea for the project, designed the study and undertook the interviews and participant observation. NB assisted in designing the study and acquiring and co-ordinating data collection. MN assisted in designing the study and co-ordinating data collection. HA, NB and MN contributed to drafting the manuscript and agreed on the final draft of the paper.

## Pre-publication history

The pre-publication history for this paper can be accessed here:


